# Early Intervention of Didang Decoction on MLCK Signaling Pathways in Vascular Endothelial Cells of Type 2 Diabetic Rats

**DOI:** 10.1155/2016/6704851

**Published:** 2016-09-15

**Authors:** Shoujiao Ye, Zhenqiang Song, Jing Li, Chunshen Li, Juhong Yang, Bai Chang

**Affiliations:** ^1^Endocrinology, 2011 Collaborative Innovation Center of Tianjin for Medical Epigenetics, Key Laboratory of Hormones and Development (Ministry of Health), Metabolic Diseases Hospital and Tianjin Institute of Endocrinology, Tianjin Medical University, No. 66, Tongan Road, Heping District, Tianjin 300070, China; ^2^Endocrinology, Nanyang TCM Hospital, No. 939, Qiyi Road, Wolong District, Nanyang, Henan 473000, China; ^3^Clinical Medicine Combined with Chinese Traditional Medicine and Western Medicine, Tianjin Chinese Medical University, No. 88, Yuquan Road, Nankai District, Tianjin 300193, China

## Abstract

In the study, type 2 diabetic rat model was established using streptozotocin (STZ) combined with a high-fat diet, and the rats were divided into control and diabetic groups. Diabetic groups were further divided into nonintervening, simvastatin, Didang Decoction (DDD) early-phase intervening, DDD mid-phase intervening, and DDD late-phase intervening groups. The expression level of MLCK was detected using Western Blot analysis, and the levels of cyclic adenosine monophosphate (cAMP), protein kinase C (PKC), and protein kinase A (PKA) were examined using Real Time PCR. Under the electron microscope, the cells in the early-DDD-intervention group and the simvastatin group were significantly more continuous and compact than those in the diabetic group. Compared with the control group, the expression of cAMP-1 and PKA was decreased in all diabetic groups, whereas the expression of MLCK and PKC was increased in early- and mid-phase DDD-intervening groups (*P* < 0.05); compared with the late-phase DDD-intervening group, the expression of cAMP-1 and PKA was higher, but the level of MLCK and PKC was lower in early-phase DDD-intervening group (*P* < 0.05). In conclusion, the early use of DDD improves the permeability of vascular endothelial cells by regulating the MLCK signaling pathway.

## 1. Introduction

Diabetic macrovascular complications are the leading cause of death and disability causing 70–80% of deaths in diabetic patients [1]. At present, there are many theories about the pathogenesis of diabetic vascular diseases, including insulin resistance, oxidative stress, lipid metabolism disorder, and inflammation theory. According to these theories, many medications have been developed to treat diabetic vascular diseases. However, these medications are not good enough with the limited effect or adverse effect; therefore we used the traditional Chinese medicine to treat this disease. We have found that DDD combined with oral hypoglycemic agents significantly increases the diastolic rate of brachial artery blood flow as well as serum NO expression and decreases plasma ET-1 levels, therefore repairing endothelial cell injury and delaying the progress of vascular disease in diabetic patients [2]. In another study, we confirmed that early intervention with DDD can significantly increase the serum levels of IL-4 and IL-3 and reduce the expression of TNF-*α*, MCP-1, CD68, and E-selectin in the aorta, thereby inhibiting the inflammatory injury and delaying the development of diabetic vascular disease in type 2 diabetic rats [3, 4]. This study investigated the effect DDD on the permeability of vascular endothelial cells when used early and discusses its roles in improving endothelial cell damage and its promise in the prevention of diabetic macrovascular complications.

## 2. Materials and Methods

### 2.1. Reagents

Streptozotocin (STZ) was obtained from Sigma (USA); glucometer and blood glucose test strips were obtained from ACCU-CHEK Performa; Real Time PCR kits were obtained from Beijing CWbio Co. Ltd.

Raw DDD herbs including 6 g rhubarb, 10 g leech, 10 g peach seed, and 10 g gadflies were purchased from the Pharmacy Department of the First Teaching Hospital of Tianjin University of Traditional Chinese Medicine. According to the traditional preparation of a water decoction, all the herbs were boiled in water for 1 hour, 3 times; the filtrates were collected and concentrated to 1 g/mL (rude herbs) under reduced pressure and then stored at 4°C.

Simvastatin tablets were obtained from Hangzhou MSD Pharmaceutical Co. Ltd. (20 mg/tablet, batch number H19990366).

### 2.2. Animals and Grouping

150 healthy Sprague Dawley (SD) male rats aged one month (body weight 195.99 g ± 15.10 g) were provided by the Tianjin Experimental Animal Center (qualification certificate number SCXK army 2014-0001); after one week of adaptive feeding, 150 SD male rats were randomly divided into control (20 rats) and diabetic (130 rats) groups. The rats in the control group were feed with a normal chaw diet, whereas rats in the diabetic groups were feed with a high-fat diet. After 12 weeks, blood from the angular vein was taken, and fasting blood glucose (FBG) and serum insulin (INS) were determined. The insulin resistance index (ISI) was calculated by using the following steady state model evaluation method; ISI = ln⁡[(FBG)(Fins)]^−1^. After the appearance of insulin resistance, all rats in the diabetic groups were injected intravenously with 30 mg/kg streptozotocin (STZ) to induce diabetes, whereas rats in the normal control group were injected with the same dose of citric acid buffer. Diabetes was determined to be successfully established if the blood glucose level was higher than 16.7 mmol/L 3 days after STZ injection. Diabetes was induced successfully in 108 rats (80%). Except for rats in the DDD early-phase intervening group, all other diabetic rats were randomly subdivided into diabetic nonintervening, simvastatin, DDD mid-phase intervening, or DDD late-phase intervening groups.

### 2.3. Intervention and Specimen Acquisition

DDD was initiated 4 weeks before diabetes was induced in the DDD early-phase intervening group. DDD was initiated upon induction of diabetes in rats in the DDD mid-phase intervening group. DDD was initiated 4 weeks after diabetes was induced in the DDD late-phase intervening group. The dosage of DDD was 2.3 g/(kg·d) based on the equivalent dose for humans. Rats in the simvastatin group were administered 0.16 g/(kg·d) simvastatin calculated according to 6.3 times of the human clinical dose; rats in the control group and the diabetic nonintervening group were given the same amount of sterile water. Fasting blood glucose and postprandial blood glucose were detected every 2 weeks. The animals were sacrificed at 24 weeks. Thoracic aorta tissue was cryopreserved in liquid nitrogen for later analysis.

### 2.4. Biochemical Indicators and Detection Methods

#### 2.4.1. Western Blot

**Figure 1:**
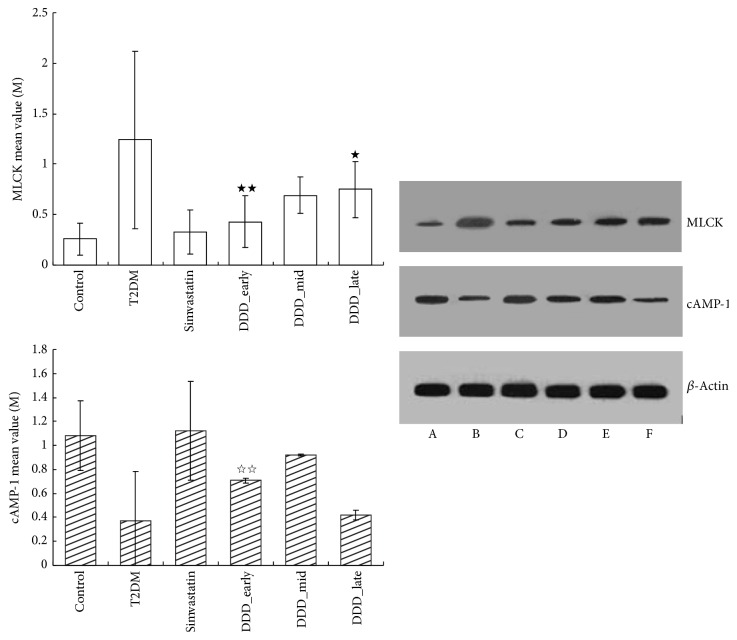
The protein level of MLCK and cAMP-1 in type 2 diabetic rats intervened by different medicines. ^★★^ *P* < 0.01 versus T2DM and ^★^ *P* < 0.05 versus DDD_early. ^☆☆^ *P* < 0.01 versus T2DM and ^☆☆^ *P* < 0.01 versus DDD_late. The expressions of MLCK and cAMP-1 in the early-phase DDD-intervening group exceed those of the late-phase DDD-intervening group (*P* < 0.05). A, control group; B, T2DM; C, simvastatin group; D, DDD early-phase intervening group; E, DDD mid-phase intervening group; F, DDD late-phase intervening group.

The expression level of myosin light chain kinase (MLCK) and cyclic adenosine monophosphate (cAMP-1) of the thoracic aorta was detected using Western Blot analysis. Rat aortic tissue was collected, centrifuged, washed, and cleaned with a cleaning solution. The total protein of the aorta tissue was extracted and quantified using Coomassie blue. The proteins were separated by denaturation gel electrophoresis and then incubated with primary antibody (MLCK antibody dilution was 1 : 300 and loading control dilution was 1 : 1000; cAMP-1 antibody dilution was 1 : 500 and loading control dilution was 1 : 800) and horseradish peroxidase labeled second antibody (dilution was 1 : 3000). *β*-Actin expression was evaluated to confirm equal amounts of loading control using a mouse monoclonal anti-*β*-actin antibody. The average luminosity value for each band was analyzed with gel image analysis. The electrophoresis bands of MLCK and cAMP-1 are shown in Figure 1.

#### 2.4.2. Real Time PCR

Real Time PCR was used to detect mRNA levels of protein kinase C (PKC) and protein kinase A (PKA). The sequence of PCR primers is listed as follows: PKC: the length of the amplified fragment was 133 bp, upstream 5′TGGCAAGGTCATGCTCTCAG3′, downstream 5′GGAAGCAGGAATGGAGCTGA3′; PKA: the length of the amplified fragment was 80 bp, upstream 5′CGTACTTGGACCTTGTGTG3′, downstream 5′CAGCCATCTCGTAGATGA3′. The PCR products were separated by agarose gel electrophoresis and then quantified by gel image analysis. Using an ABI 7500 fluorescence quantitative PCR instrument, we analyzed the relative quantitative data using the 2^−CTΔΔ^ method.

#### 2.4.3. Immunohistochemical Staining

The expression levels of MLCK and PKC of the thoracic aorta were detected by the immunohistochemical method. The aortic endothelium of rats in experimental group and control group was fixed, encased, sliced, dewaxed, and so forth. Then the images of the positive and negative structure were collected and read under the microscope.

#### 2.4.4. Ultrastructure under Electron Microscope

The ultrastructure of the endothelial cells and endothelial intercellular connection were observed under the electron microscope.

### 2.5. Statistical Methods

SPSS 17 statistical software was used for analysis. All data are expressed as the mean and the standard deviation (mean ± SD). The difference between two groups was compared by the independent sample* t*-test. The difference between the 7 groups was analyzed by the single factor analysis of variance (ANOVA one-way); difference between two groups was analyzed by the LSD method if the homogeneity of variance was satisfied, and Dunnett's T3 method was used if the homogeneity of variance was not satisfied; the test level was *α* = 0.05.

## 3. Results

### 3.1. The Blood Glucose and Insulin Levels in Different Groups

**Figure 2:**
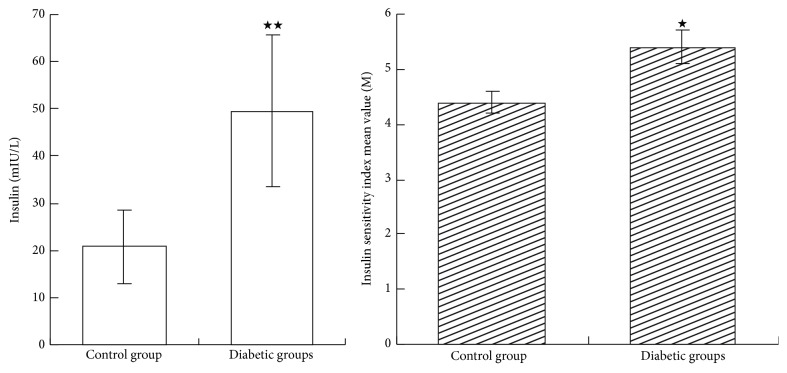
Insulin and insulin sensitivity index at 12th week of this experiment. Values are means ± SD in each group. ^★★^ *P* < 0.01 versus control group and ^★^ *P* < 0.05 versus control group. No significance was found in blood glucose between diabetic groups (*P* > 0.05).

**Figure 3:**
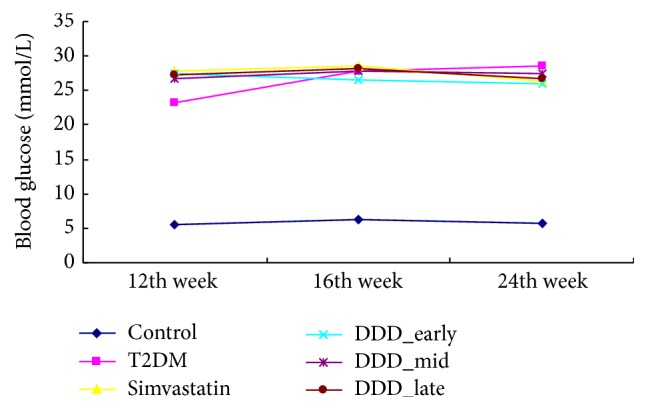
Random blood glucose in different groups (mean ± SD). No significance was found in blood glucose between diabetic groups (*P* > 0.05). T2DM: diabetes with no intervention group; DDD early group: T2DM intervened by DDD at early phase; DDD mid group: T2DM intervened by DDD at mid phase; DDD late group: T2DM intervened by DDD at late phase.

As shown in Figure 2, at 12 weeks, compared with the control group, rats in the diabetic groups had significantly higher levels of blood glucose (*P* < 0.05) but lower levels of the insulin sensitivity index (*P* < 0.05). The results of blood glucose are shown in Figure 3 and no significant differences were found in blood glucose levels between diabetic groups (*P* > 0.05).

### 3.2. Effects of DDD-Intervening Groups on the Expression of MLCK, cAMP-1, PKC, and PKA in Aorta Tissues of Type 2 Diabetic Rats

**Figure 4:**
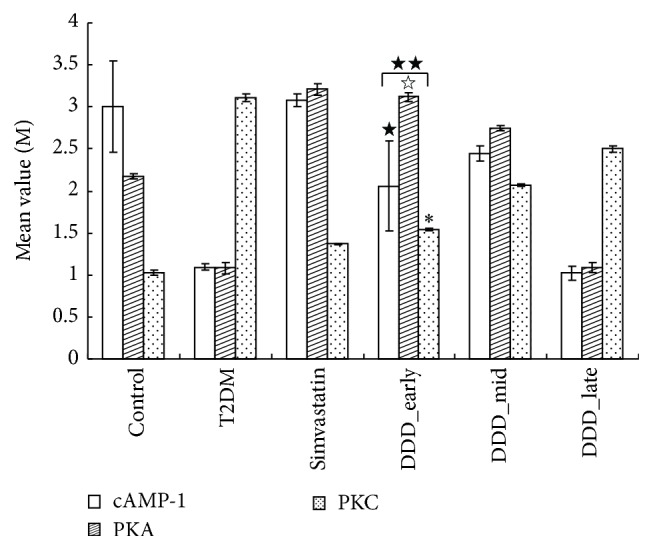
Effects of Didang Decoction on the expression of mRNA gene transcription level on cAMP-1, PKA, and PKC (mean ± SD). ^★★^ *P* < 0.01 versus T2DM and ^★^ *P* < 0.05 versus DDD_late. ^☆^ *P* < 0.05 versus DDD_late. ^*∗*^ *P* < 0.05 versus DDD_late and ^*∗*^ *P* > 0.05 versus control.

As shown in Figures 1 and 4, compared with the control group, the expression of cAMP-1 and PKA was decreased in all diabetic groups, whereas the expression of MLCK and PKC was increased (*P* < 0.05). Compared with the T2DM nonintervening group, the expression of cAMP-1 and PKA was increased, but the expressions of MLCK and PKC were decreased in the early- and mid-phase DDD-intervening groups (*P* < 0.01); the expressions of PKA, MLCK, PKC, and cAMP-1 in the early-phase DDD-intervening group were better than those of the late-phase DDD-intervening group (*P* < 0.05).

**Figure 5:**
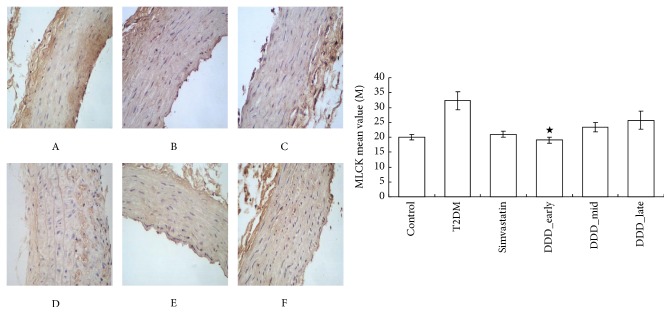
The expression level of MLCK of the thoracic aorta was detected by the immunohistochemical method (mean ± SD). A, control group; B, T2DM; C, simvastatin group; D, DDD early-phase intervening group; E, DDD mid-phase intervening group; F, DDD late-phase intervening group. ^★^ *P* > 0.05 versus control and simvastatin and ^★^ *P* < 0.05 versus DDD_late.

**Figure 6:**
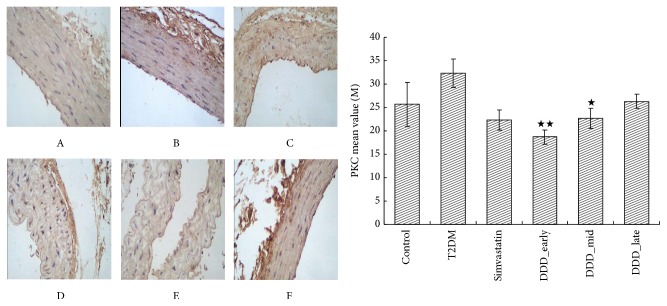
The expression level of PKC of the thoracic aorta was detected by the immunohistochemical method (mean ± SD). A, control group; B, T2DM; C, simvastatin group; D, DDD early-phase intervening group; E, DDD mid-phase intervening group; F, DDD late-phase intervening group. ^★★^ *P* < 0.01 versus T2DM and ^★^ *P* > 0.05 versus simvastatin.

As shown in Figures 5 and 6, compared with the T2DM nonintervening group, the expression of MLCK and PKC was decreased in DDD-intervening groups (*P* < 0.01). Compared with the late-phase DDD-intervening group, the expression of MLCK and PKC was decreased in early-phase DDD-intervening group (*P* < 0.05).

### 3.3. The Effect of DDD on the Endothelial Cells in Aortas of Diabetic Rats

**Figure 7:**
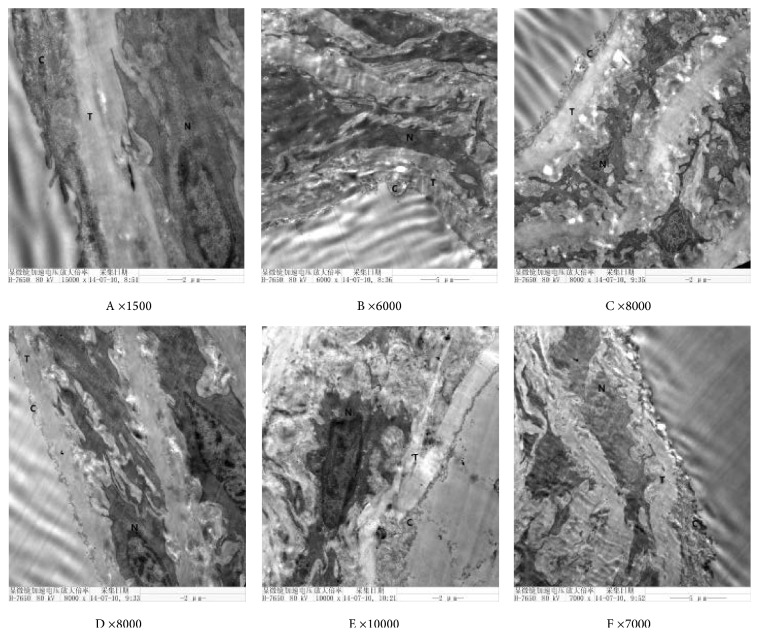
Effect of Didang Decoction on the ultrastructure of vascular endothelial cells and intercellular connections in rats with diabetes mellitus. A, control group; B, T2DM; C, simvastatin group; D, DDD early-phase intervening group; E, DDD mid-phase intervening group; F, DDD late-phase intervening group. C, endothelial cell; T, elastic membrane; N, smooth muscle.

The results of electron microscopy for the control group show a smooth cord-like connection between endothelial cells. The connection in the early-phase DDD-intervening group and the simvastatin group was continuous and compact, significantly better than those in the other diabetic groups. In the diabetic group, the majority of the tight junctions between the two endothelial cells were opened. The density of the cell membrane on both sides was decreased, the cell profile was not clear, and the nuclei were loose to different degrees. Electron microscope images of these phenomena are shown in Figure 7.

## 4. Discussion

Macrovascular disease is one of the main complications of type 2 diabetes, and atherosclerosis is the main pathological change. Our previous study has found that DDD is efficient in the treatment of diabetic patients. This study investigated the effect of early intervention with DDD on the expression of MLCK, cAMP-1, PKC, and PKA and gene expression in vascular endothelial cells of aortas of diabetic rats, to explore the roles of DDD on the permeability of vascular endothelial cells and its relationship with the MLCK signaling pathway.

The main vascular endothelial cell permeability modulation is contraction caused by the interaction of actin and myosin. When the contraction is greater than the adhesion, vascular endothelial cell permeability is increased [5]. MLCK plays an important role in modulating endothelial cell permeability. MLCK is a Ca^2+^/calmodulin-dependent complex. MLCK modulates endothelial cell permeability mainly by phosphorylating the myosin light chain (MLC), which then activates the myosin heavy chain ATP enzyme. Activating the ATP enzyme produces the energy to enhance the interaction of myosin and actin, causing cell contraction and cell junction changes, which then leads to increased permeability [6, 7]. Recent studies [8] have indicated that protein kinase C (PKC) is involved in the regulation of MLCK phosphorylation. Some studies suggest that [9] PKC phosphorylation may change MLCK activity and eventually cause MLC phosphorylation. In addition, PKC plays a role in increasing the level of cGMP and NO by the phosphorylation of S1179 in eNOS. The increased level of cGMP increases the level of cyclic adenosine monophosphate (cAMP) [10]; however, almost all the effects of cAMP-1 in the cell are accomplished by activating PKA, which then phosphorylates its substrate protein. It has been shown that [11, 12] increasing the concentration of intracellular cAMP-1 can upregulate the activity of PKA. The PKA phosphorylation site has been confirmed in MLCK of endothelial cells; therefore, cAMP-1 can inhibit the activity of MLCK by the activation of PKA and regulate vascular endothelial permeability [13]. Studies have shown that [14] the phosphorylation of myosin light chain induces the concentric contraction of vascular smooth muscle cells, resulting in the increased thickness of the intima of vascular smooth muscle cells and the formation of a large number of foam cells, leading to the occurrence of atherosclerosis. Therefore, MLCK in arterial endothelial cells and smooth muscle cells plays a key role in the occurrence and development of diabetic macrovascular disease.

Statins are the most commonly used medicine in clinic to treat dyslipidemia at present. They can prevent the occurrence and development of atherosclerosis effectively. Statins in addition to their lipid-lowering effect can also improve inflammation and oxidative stress, promote angiogenesis formation, improve the bioavailability of the nitric oxide (NO), repair the damaged vascular endothelial cells, and stabilize and reverse atherosclerotic plaque. Simvastatin is used as the positive control for diabetic vascular disease in this research because of its pleiotropic effects.

DDD originates from* Treatise on Cold Pathogenic Diseases*, which is mainly composed of leech, gadfly, and peach seed, and can improve blood circulation and remove obstructions in collaterals, as well as eliminate blood stasis by catharsis. Many studies have shown that leech and hirudin have an antithrombotic effect. Emodin has the effect of anticoagulation and promotion of blood circulation, whereas Rheochrysin inhibits platelet aggregation; both Emodin and Rheochrysin are in rhubarb. The alcohol extract of peach seed has an anticlotting effect, and its glycerol trioleate has anticoagulant activity. In Chinese medicine, diabetic macrovascular disease is classified as “arthromyodynia,” “flaccidity diseases,” and so forth. The basic pathogenesis of this disease includes Yin deficiency and hot continually thirsty and wasting, disharmony of Qi and blood, fluid depletion, and blood stasis, accompanied by blood heat and blood stasis. At this stage the treatment is difficult. As the* Inner Canon of Yellow Emperor* (one of the four classics of TCM) said, “when a person has beening [*sic*] feel thirsty then began to dig a well or the war has beening [*sic*] begun that the soldiers began to making weapons.” It means that something had happened but you have not prepared for it, just like the disease is serious to find a doctor. It is too late to treat diseases such as this, so traditional Chinese medicine pays more attention to the prevention of diseases and emphasizes boosting the body's defense capabilities in the early stages of disease. Here, we comply with the principles of “the prevention theory” and use DDD in the early phase of diabetic macrovascular diseases to explore its improvement of the damaged tissues as well as its protective roles in exempting these tissues from further damage caused by many pathogenic factors.

## 5. Summary

We used high-fat diet followed by the small dose of STZ to establish type 2 diabetic animal model in this experiment. The high-fat diet followed by small dose of STZ induced the obvious pathological changes in the blood vessel endothelium of diabetic rats. The finding suggested that the traditional Chinese medicine DDD intervention, particularly early intervention of DDD, significantly ameliorated the injury of vascular endothelium, regulated intercellular junctions of endothelial cells, changed the permeability of vascular endothelial cells, and eventually prevented vascular atherosclerosis. Further mechanisms of investigation indicated that the effect of DDD intervention is related to upregulating the expression of cAMP-1 and PKA and downregulating the expression level of MLCK and PKC. Thus DDD may delay the progression of diabetic macroangiopathy.

The regulation of cell permeability of vascular endothelial cells is a complex process. In this study, we found a significant improvement of vascular endothelial cell permeability after early intervention with DDD. However, our study has some limitations; we did not quantify the permeability of endothelial cell; moreover, further studies need to address the multiple roles and mechanism of DDD in the prevention and treatment of diabetic macrovascular disease.
